# Mapping evidence on the impact of junk food on anaemia among adolescent and adult population: a scoping review

**DOI:** 10.1186/s40795-025-01079-1

**Published:** 2025-05-14

**Authors:** Joyce Sangeetha Soans, Judith Angelitta Noronha, Suneel C Mundkur, Baby S. Nayak, Meenakshi Garg, Roshan David Jathanna, Edlin Glane Mathias

**Affiliations:** 1 Department of OBG Nursing, Manipal College of Nursing, Manipal Academy of Higher Education, Manipal, Karnataka India; 2 Department of Paediatrics, Katurba Medical College, Manipal Academy of Higher Education, Manipal, Karnataka India; 3 Department of Child Health Nursing, Manipal College of Nursing, Manipal Academy of Higher Education, Manipal, Karnataka India; 4 Department of Dietetics and Applied Nutrition, WGSHA, Manipal Academy of Higher Education, Manipal, Karnataka India; 5 Department of Computer Science, Manipal Institute of Technology, Manipal Academy of Higher Education, Manipal, Karnataka India; 6 Department of Health Technology and Informatics, Prasanna School of Public Health, Manipal Academy of Higher Education, Manipal, Karnataka India

**Keywords:** Anaemia, Junk food, Adolescents, Consumption, Wellbeing

## Abstract

**Background:**

Anaemia is a significant global health issue, with adolescents being a particularly vulnerable group. In developing countries, 27% of adolescents are affected by anaemia, compared to a much lower prevalence of 6% in developed countries. This scoping review aims to investigate the intake of junk food and the development of anaemia, providing a foundation for future research in this field.

**Methods:**

A systematic search was conducted across Scopus, PubMed, EBSCO, CINHAL, WOS and ProQuest using specific keywords. Inclusion criteria comprised all quantitative studies examining the association between nutrition and the development of anaemia. Articles selected for analysis were restricted to those published in English Language between 2014–2024 and available as full-text articles.

**Results:**

Among the articles that were screened, 20 articles met the criteria for data extraction. Four studies did not reveal statistically significant correlations between nutrition and the development of anaemia, while two studies provided evidence for significant associations. The findings indicated increased anaemia was associated with (a) fast food intake, western pattern of diet, poor eating habits, omission of breakfast and (b) diminished consumption of fruits and vegetables, iron intake, seafood, nuts, and seeds.

**Conclusion:**

The existing evidence suggests a link between the consumption of junk food and the prevalence of anaemia among adolescents. However, there is a lack of comprehensive studies that thoroughly explore this connection. This gap in research underscores the urgent need for more in-depth studies to understand how modifiable risk factors like junk food consumption contribute to anaemia in adolescents, with the goal of improving prevention and management strategies. Addressing this issue aligns with Sustainable Development Goal (SDG) 3, which aims to ensure healthy lives and promote well-being for all at all ages, which focuses on ending preventable deaths of children and addressing adolescent health. Additionally, this research also contributes to end hunger, achieve food security, and improve nutrition.

**Supplementary Information:**

The online version contains supplementary material available at 10.1186/s40795-025-01079-1.

## Background

Anaemia is among the most prevalent nutritional disorders worldwide, affecting approximately one-third of the global population, largely due to iron deficiency. According to the World Health Organization, nearly two billion people, or about 25% of the global population, are affected by anaemia, with approximately half of these cases resulting from iron deficiency anaemia (IDA) [1]. Among adolescents, the most significant risk factors for IDA include poor food intake practices, female gender, menstruation, parasitic infections, inadequate educational qualifications [2], and low economic status [3].

Iron deficiency is the leading predictor of anaemia, followed by other factors such as haemoglobinopathies, vitamin A deficiency, and zinc deficiency [4]. Malnutrition, which includes both undernutrition and over nutrition, plays a crucial role in the development of anaemia. In South Asian countries, one in three people is malnourished, driven by poor diet quality, inadequate healthcare, and broader socio-economic factors like political instability, low economic development, and inequality [5].

Anaemia adversely affects cognitive function in adolescents by disrupting neurotransmitter production, delaying brain development, and impairing cognitive processing. Addressing anaemia through proper nutrition and medical treatment is essential for supporting healthy cognitive development during this crucial stage of life [6].

Dietary deficiencies are a major cause of anaemia, with cultural practices and perceptions about diet significantly influencing food choices. Research on adolescent girls has shown that while there is some awareness that poor diet can lead to anaemia, there is often limited knowledge about the specific dietary elements necessary to prevent it [7]. Junk food consumption exacerbates this issue by displacing nutrient-rich foods in the diet, leading to further nutritional deficiencies [8]. Adolescents, in particular, are prone to consuming snacks made from refined cereals and carbonated drinks, while showing a lower inclination towards fruits and vegetables, which are essential sources of iron and other vital nutrients [9].

The widespread consumption of junk food, which provides empty calories but lacks essential nutrients like iron, vitamin A, and zinc, directly contributes to the development of anaemia [8]. Popular foods like street snacks with black rock salt, soft drinks, and pre-packaged spice mixes often lack the essential nutrients required to support healthy iron levels, which can elevate the risk of anaemia [10]. Furthermore, IDA is a preventable and treatable condition, but it often goes unaddressed due to poor dietary habits and a lack of awareness about the importance of nutrient-rich foods [1, 6].

This review aims to explore the overlooked relationship between junk food consumption and the development of anaemia, particularly in adolescents. By identifying gaps in the existing literature, this review will shed light on the nutritional deficiencies caused by junk food and their contribution to the rising incidence of anaemia. Understanding these gaps are crucial for developing targeted interventions and public health strategies to combat anaemia, especially in vulnerable populations such as adolescent girls.

## Methods and Materials

This review followed a scoping review methodology, we explored evidence to gain a deeper understanding on consumption of junk food to anaemia. This scoping review is reported according to the “Preferred Reporting Items for Systematic Reviews and Meta-Analyses (PRISMA) Extension for Scoping Reviews Checklist” [11].We adopted the six-stage methodological framework of scoping review by Arksey and O’Malley [12].

The framework consists of the following steps.

### Step 1: Specify the research questions

The following research questions were formulated.

What is the impact of junk food consumption on the development of anaemia among adolescents?

We followed the Population, Concept, Context, and Study design criteria for identifying the studies.

#### Population

We included adolescents and young adults aged 10 to 25 years. This review encompasses studies on consumption, intake, and dietary patterns, as well as sociodemographic factors associated with anaemia. Additionally, we included studies on risk factors, eating habits, and the impact of fast food and junk food consumption.

#### Concept

Junk food refers to foods that are high in calories, fats, sugars, and/or salt but low in essential nutrients such as vitamins, minerals, and fibre [13]. These foods are typically highly processed and may include items like fast food, sugary snacks, sodas, and pre-packaged treats [14]. Regular consumption of junk food has been associated with various negative health outcomes, including obesity, cardiovascular diseases, and nutritional deficiencies [15, 16].

#### Context

The existing literature shows inconsistent findings regarding the relationship between junk food consumption and anaemia. To gain a comprehensive understanding of this issue, we included studies conducted across various global contexts, encompassing low, middle, and high-income countries. These studies were drawn from university, hospital, and community settings, regardless of geographical conditions.

#### Study designs

We included studies that reported on the relationship between junk food and anaemia. This review encompassed quantitative studies of all designs (Randomized controlled trials, non-randomized controlled trials, observational, cross-sectional, and cohort). We excluded protocols, editorials, social media posts, and magazine reports, as they do not provide substantial information for synthesis. Records without available full texts were also excluded, and only literature published in English was considered.

### Step 2: Identify the Relevant Literature

An extensive search was carried out to identify relevant studies. A search strategy was formulated by extracting keywords such as ("Eating"[Mesh] OR “Dietary intake” OR eating OR intake OR Consumption) AND (Junk food OR fast food OR food preserved OR frozen foods OR candy OR canned OR convenience food OR ultra- proceeded foods OR ultra-processed foods) AND (Haemoglobin OR Anaemia OR “Iron deficiency anaemia” OR Anaemia) AND ("Adolescent"[Mesh] OR adolescence OR teen OR teens OR teenager OR youth OR youngsters OR youthful) from the Medical Subject Heading (MeSH) browser [17] and consulting with subject-matter experts (JAN, SCM), as well as reviewing relevant literature. To ensure thorough coverage, six electronic databases PubMed (NCBI), CINAHL (EBSCO), Embase (Elsevier), Web of Science (Clarivate), ProQuest, Scopus (Elsevier), and Cochrane Library were searched on 31/07/2024 by SJS, with further validation by JAN. The search strategy utilized Boolean operators like"AND"and"OR"to combine terms. Studies published between January 2014 and July 2024 were included in this review. A detailed search strategy is provided in Appendix 1.

### Step 3: Selection of Studies

The database search results were imported into Rayyan software for analysis. [18]. After removing duplicates, two reviewers (SJS and JAN) independently screened the titles and abstracts of the studies. Any discrepancy between the selection were resolved through a consensus-building process. Following these the same two reviewers (SJS and JAN) independently assessed the full texts. In case of disagreements, it was resolved through discussion and in consultation with Third reviewer (MG). Since this is a scoping review focused on providing an overview of the literature, the quality assessment and risk of bias for the included studies were not conducted, meaning that these factors did not influence the results or their interpretation.

### Step 4: Charting the Data

Five reviewers independently extracted data independently (SJS, JAN, SCM, BSN, MG). The data quality was ensured by crosschecking the extracted data by two reviewers (SJS and EGM). Relevant data on authors, country/region, study designs, objectives, methods, key findings and conclusion on consumption of junk food to anaemia were extracted using a predesigned data extraction form on Microsoft Excel.

### Step 5: Collecting, Summarizing, and Reporting Results

The findings were summarized using narrative strategy supported by tables where applicable. The results were presented as authors, study year, aims type of study, Methods, key findings and conclusion. The junk food and consumption (type of junk food, frequency of consumption, quantity consumed) and anaemia indicators like prevalence and risk factors of anaemia and association between junk food and anaemia (Table 1).

**Table 1:** Characteristics of the included studies

Author name & year	Aim	Study design	Country	Sample size and Age	Data collection methods	Key findings	Conclusion
Batool Zaira, Aziz Karim Saadiya, Begum Amna, 2017	To investigate the frequency of anaemia and its socioeconomic and dietary determinants among adolescent girls	Cross sectional	Pakistan (Karachi)	N = 497 13–18 years	Interview method using questionnaire on socio-economic, dietary, and demographic profiles Clinical examination for signs of anaemia was conducted Haemoglobin estimation was performed using a portable analyser The dietary recall was collected using a questionnaire tailored for the study, which comprised daily, and weekly food item recalls and posed questions regarding the quality of food consumed on a weekly basis Anthropometric measurement was done by measuring the height and weight to calculate the BMI	36.5% participants were anaemic with haemoglobin levels below 11 g/dL Anaemia was significantly associated with lower socioeconomic status, parental education, and dietary intake Anaemic girls had significantly lower consumption of eggs, milk, spinach, green leafy vegetables, and chocolate/candies compared to non-anaemic girls	Improving socioeconomic conditions and implementing dietary interventions that promote iron-rich foods are crucial for reducing the prevalence of anaemia among adolescent girls in rural Pakistan
Ma Jie et.al 2022	To explore the association between dietary patterns and explore, their association with anaemia among rural children	Cross sectional	China	N = 1476 9–16 years Boys—787 Girls—689	The survey comprised of a questionnaire survey, physical examination, and laboratory examination Factor analysis identified four dietary patterns with anaemia risk among children in rural areas Anaemia diagnosed based on haemoglobin using WHO criteria Nutritional status assessed using BMI –for-age reference and growth retardation Dietary patterns were constructed using principal component analysis and factor rotation	A fast-food consumption pattern was associated with an increased risk of anaemia in children (PR = 1.767), particularly in girls after menarche and those who were underweight A dietary pattern rich in meat and eggs was linked to a lower risk of anaemia in children (PR = 0.498) Dietary patterns significantly impacted the prevalence of childhood anaemia in rural areas Anaemia prevalence was 10.4% higher in girls compared to boys	Fast food consumption has been identified as a contributing factor to the risk of anaemia in children, while a diet rich in meat and eggs has shown protective effects, particularly in those entering puberty. The findings of this study can be used to develop evidence-based nutritional interventions to address anaemia in children
Guadalupe Arli, et. al 2006	To describe the dietary patterns (DPs) and to examine its association with malnutrition indicators	Cross-sectional	Mexico Urban and rural areas	N = 7380 12–19 years Boys: 12–15 years,16–19 years Girls: 12–15 years, 16–19 years Boys—4,574 Girls—2,222	This study used data from the National Health and Nutrition Survey (ENSANUT-2006) Principal component analysis derived four dietary patterns Dietary patterns were associated with nutritional status using prevalence ratio Seven-day FFQ with 101 food items for dietary data collection Anthropometric techniques for measuring weight and height Haemoglobin concentrations adjusted for residential altitude	Among children with anaemia, approximately 30% were overweight or obese Adolescents with higher adherence to the Western dietary pattern had a greater prevalence of overweight and obesity (PR: 1.15, 95% CI: 1.08–1.21) A higher prevalence of anaemia was observed among those following the Western dietary pattern (PR: 1.18, 95% CI: 1.03–1.35) The non-traditional and breakfast-type dietary pattern was inversely associated with anaemia in younger adolescents	The Western dietary pattern was positively associated with both obesity and anaemia in adolescents Nutritional guidance should focus on addressing all forms of malnutrition Adolescence is a critical period for shaping long-term health and dietary habits, emphasizing the need for early intervention and education
Akhtar Naveed, Zareen Humaira, Sarmad Rubina 2018	To assess the dietary habits, nutritional status and their association among female medical students	Cross-sectional descriptive study	Pakistan (Medical college of Lahore)	N = 114 19–21 years	Structured questionnaire used to collect data on dietary habits Haemoglobin levels were tested using Sysmex Mid upper arm circumference (MUAC) was measured with a measuring tape Body mass index (BMI) was measured using a weighing machine	36.8% of participants were underweight, and 36% were anaemic 94.7% preferred traditional food, and 73.3% consumed breakfast daily 64% regularly consumed fast/junk food, while 71.1% ate meat at least three times per week Eating habits significantly influenced nutritional status, with skipping breakfast linked to anaemia A positive association was found between eating habits and haemoglobin levels (p = 0.001) Regular breakfast intake was positively associated with BMI and MUAC (p = 0.003, p = 0.02) and overall nutritional status (p = 0.003)	Eating habits significantly impact nutritional status, independent of socio-demographic characteristics. Poor eating habits are associated with anaemia, and skipping breakfast contributes to malnutrition
Marwan Jalambo et al. 2015–2016	Determine the prevalence and identify the risk factors associated with anaemia, iron deficiency, and iron-deficiency anaemia	Cross-sectional descriptive study	Palestine (Gaza Strip)	N = 330 15–19 years	Data was collected through Three types of questionnaires: FFQ, sociodemographic, sedentary behaviour and physical activity Food frequency questionnaire (FFQ) was utilized for dietary assessment Sociodemographic questionnaires were administered to gather background information Questionnaires were used to collect the sedentary behaviour and physical activity Anthropometric measurements were conducted for physical assessment Blood analysis was performed to determine iron status	Anaemia was present in 35.8% of female adolescents, iron deficiency (ID) in 40.3%, and iron deficiency anaemia (IDA) in 26.0% ID and anaemia were not significantly associated with physical activity (P < 0.05) ID was linked to poor dietary habits, such as skipping breakfast and frequent junk food consumption IDA was associated with low fruit and vegetable intake among female adolescents Mother's education was associated with ID but not with other socio-demographic factors Reduced meat intake increased the likelihood of anaemia (OR = 0.95, 95% CI: 0.92–0.96, P < 0.001) Anaemia in female adolescents was associated with lower intake of meat, milk products, and certain beverages	A significant association was found between iron deficiency (ID), anaemia, and iron deficiency anaemia (IDA) and dietary behaviours, including skipping breakfast and consuming large amounts of junk food, particularly beverages such as soft drinks, artificial juices, coffee, and tea Additionally, increased intake of milk and legumes was linked to a higher risk of ID and IDA in these demographics
Hariyanto, Novan et.al, 2022	The purpose of this study was to analyse the relationship between diet pattern and the incidence of anaemia in teenage girls	Cross sectional	Indonesia	N = 98 15–49	Food frequency questionnaire model used to assess diet patterns Haemometer and observation sheet are used to determine haemoglobin levels and record additional relevant data	The overall prevalence of anaemia in Indonesia was 21.7%, with rates of 26.4% among adolescents and 50–60% among young women 88.8% of participants had poor eating habits, while only 11.2% followed a good dietary pattern 58.2% of individuals were not anaemic, whereas 41.8% experienced anaemia A significant negative correlation was observed between dietary patterns and anaemia (r = −0.302, p = 0.003)	Inappropriate eating patterns can contribute to anaemia, posing significant health risks for teenagers. To prevent anaemia, adolescents should reduce fast food consumption, adopt a nutritious diet rich in fruits and vegetables, and improve their knowledge and awareness of healthcare. These measures can help reduce the risk of anaemia and support overall well-being during this crucial developmental stage
Gultom Yohana, Aritonang Evawany 2020	Correlate nutrition knowledge and eating pattern with anemia incidence in female teenagers	Descriptive study with cross-sectional design	Indonesia	N = 100 15–64 years	Data obtained through haemoglobin level examination, questionnaires, food recall forms Digital acute check for haemoglobin content examination Questionnaire to assess the knowledge and eating habits 24 h diet recall forms for assessing the dietary intake Meal frequency form to tract eating patterns	The prevalence of anaemia among female teenagers in the rural area is 58% Inadequate iron intake increased the risk of anaemia by eight times The majority of female teenagers had sufficient protein intake	Female teenagers with insufficient iron intake are eight times more likely to develop anaemia compared to those with adequate intake Anaemia can result from multiple nutrient deficiencies, and infectious diseases may further hinder iron absorption Ensuring proper nutrition and a supportive environment is essential for promoting healthy physical and psychological development during adolescence
Parveen Nisha, Rani Seema, John Neha, 2018	To assess the prevalence of anaemia and identify dietary practices among adolescent girls	Descriptive survey	India	N = 100 Girls- 100 Adolescents aged 11–17 years were included in the study	Anaemia was assessed using flow cytometry and the SLS-haemoglobin method The data on balanced diet behaviour was collected through a structured questionnaire and recording sheets	66% of adolescents were anaemic, with 31% mild and 25% moderate. 10% were classified as severely anaemic A Significant relationship was found between anaemic status and the frequency of eating junk food	The high prevalence of anaemia among adolescents necessitates emphasis on iron and folic acid supplementation, iron-rich food intake, health education on personal hygiene, and periodic deworming to reduce the burden of anaemia among adolescent girls
Singh Parul, Tiwari Harish C, Sampriya Arushi, Srivastava Arun K Srivastava Dhirendra K. 2020	To estimate the prevalence of anaemia among school going adolescent girls and to study specific factors like sociodemographic factors, dietary factors and menstrual factors	Cross sectional study	India	N = 430 Girls- 430 10–19 years	Pre-phrased questionnaire was used for data collection Interview methods were used to collect the data regarding the sociodemographic variables, dietary variables, and menstruation-related variables The Anthropometric assessment of the study participants involved the measurement of height, weight, and body mass index (BMI) calculation of BMI, Height was measured using a portable stadiometer BMI calculation and haemoglobin estimation using portable instruments	Anaemia was observed in 61.39% of school-going adolescent girls, with a higher prevalence among obese individuals (66.66%) Risk factors for anaemia included age, low socio-economic status, dietary habits (intake of junk food). and menorrhagia A Strong association found between IFA intake and anaemia prevalence No significant association was observed between BMI and haemoglobin levels in the study group Factors such as religion, parental education and occupation, birth order, deworming, milk intake, jaggery intake, menstrual irregularities, polymenorrhea, and dysmenorrhea were not significantly associated with anaemia	Anaemia is highly prevalent (61.39%) among adolescent girls. Significant risk factors include age, living in a nuclear family, and low socio-economic status. Dietary factors contributing to anaemia include low intake of citrus fruits and green leafy vegetables, along with high consumption of junk food
Paramastri Rathi et al., 2015	Investigate dietary patterns, lifestyle, nutritional status, and anaemia related biomarkers among adults	Observational study	Taiwan	N = 118,924 20–45 years Boys- 43,055 Girls- 75,869	Self-reported questionnaire for information on sociodemographic data, lifestyles, medical history, and dietary habits Body weight and height were measured by using an auto-anthropometer. Weight status was defined by using BMI criteria in Taiwan, and waist circumference was used to define central obesity Biochemical data, including anaemia related biomarkers such as haemoglobin, haematocrit, and RBC were done. The level of CRP was measured using an auto-analyser. Anaemia was classified based on WHO criteria Dietary pattern derived by Reduced Rank Regression method associated with anaemia risk Demographic and lifestyle variables analysed for anaemia and biomarkers	A High dietary pattern (processed foods, and sugary beverages) increased the risk of anaemia by 59% Alcohol consumption (46%) and abnormal weight status, including underweight (20%), overweight (23%), and obesity (34%), were associated with an increased risk of anaemia The anaemia-inflammation dietary pattern was linked to a higher risk of anaemia in Taiwanese adults	Adherence to the anaemia-inflammation dietary pattern is associated with an increased risk of anaemia in Taiwanese adults. Additionally, abnormal weight status and alcohol consumption are correlated with a higher risk of developing anaemia
Ramya Neelakanda et al. 2020	To study the magnitude of anaemia in young females and its association with diet pattern	Cross sectional study	India	N = 100 Girls-100 18–40 years	Recorded demographic details, dietary patterns, and haematological parameters Obtained haematological parameters using automated cell counter and blood smears Categorized anaemia severity based on WHO criteria for haemoglobin levels	62% of participants were anaemic, with a mean age was 26.52 years Among the anaemic participants, 44% had mild anaemia, 16% had moderate, and 2% had severe anaemia 47% of those who did not consume green leafy vegetables had mild to moderate anaemia 25% of individuals who drank tea or coffee after meals had mild anaemia 14% of participants who regularly consumed junk food and 5% of those with low fruit intake were anaemic	Iron deficiency anaemia is common among young females, especially those with poor dietary habits. Health education on proper dietary practices is essential for preventing nutritional anaemia in this group
Dinar, Putri Rahmawati, Dono Indarto.,Hanim Diffah 2020	The study aimed to investigate the correlation of fast food consumption and snacking with hemoglobin (Hb) levels in female adolescents	Cross sectional study	Indonesia	N = 117 Girls −117 15–18 years	Body weight and height measured using digital scales and microtoice Fast food and snacking data collected through Food Frequency Questionnaire (FFQ) Haemoglobin levels tested using haematology analyzer at Clinical Laboratories	Fast food consumption was negatively correlated with Haemoglobin levels (r = −3.47; p = 0.001) Snacking showed no significant correlation with Haemoglobin levels (r = −1.44; p = 0.152) The prevalence of anaemia among female adolescents was 17.1%	Fast food consumption negatively impacts haemoglobin levels in female adolescents. Snacking does not show a significant correlation with haemoglobin levels. The R-square value indicates that dietary factors contribute 18.6% to haemoglobin levels
Joshi A Himanshu, Jethva J Vijay, Patel Nidhi, 2014	To evaluate the changing food pattern in adolescents and its impact on health	Observational study	India	N = 450 10–19 years	Anthropometric data was collected for Body Mass Index calculation Detailed diet history was obtained through questionnaires. Dietary habits were assessed using structured questions Consumption of junk food per week was recorded Haemoglobin concentrations were analyzed using Sahli's haemoglobin meter Socioeconomic class derived using Modified Prasad classification	23.5% of adolescents consumed junk food twice or more per week 50% of adolescents from high socioeconomic backgrounds were at risk of overweight and obesity Junk food consumption contributed to a 27.4% risk of being overweight and a 1.9% risk of obesity Anaemia was observed in 80% of females and 73.5% of males 81.5% of adolescents who consumed junk food at least twice a week were anaemic Skipping breakfast twice weekly resulted in 96.6% of adolescents being anaemic	Junk food consumption contributes to obesity, diabetes, hypertension, and coronary diseases. Reducing junk food intake can minimize health risks in adolescents
Chaturvedi Deepak, Chaudhuri K Partha, Chaudhary Anil, 2017	To study the correlation between dietary habits and anemia among adolescent girls	Analytical cross-sectional epidemiological study	India	N = 300 10–19 years	Questionnaire-cum-interview technique used for primary data collection Haematological parameters obtained using automated hematology cell counter and microscopy Dietary habits assessed through last 24 h food intake	82% participants were anaemic, with 34% having mild anaemia Despite the high anaemai rate, 91.7% of girls had a normal BMI Anaemia was more common in vegetarians and was associated with a rice-based diet compared to non-vegetarians Increased anaemia prevalence was linked to low iron and vitamin C intake There was a significant association between consuming tea or coffee after meals and the occurrence of anaemia	Adolescent girls are at a heightened risk of anemia, with severity increasing from early to late adolescence. Vegetarians, particularly those on predominantly rice-based diets, are more susceptible to anemia than non-vegetarians. Additionally, consuming tea or coffee after meals significantly correlates with reduced iron absorption, thereby increasing anemia risk
Yahya Sheherbano, et.al, 2022	To find iron deficiency anemia and the relationship between people who consumed junk food	Observational survey-based study	Pakistan	N = 200 Boys −50 Girls −150 18–35 years	Data was gathered via a questionnaire The questionnaire evaluated demographic information, nutritional status, and knowledge regarding anaemia A food frequency questionnaire checklist were used, which comprised 25 frequently consumed fast food items Measurements of Height and weight were conducted to determine Body Mass Index (BMI) Haemoglobin levels were analysed using hematology aanalyser	Anaemia awareness: 42.5% aware, 57.5% unaware 91.17% anaemic patients consumed junk food daily 85% of participants were anaemic, whereas 15% had normal haemoglobin levels BMI: 11.76% were underweight, 52.94% had normal weight, 17.65% were overweight, and 17.65% were obese A positive correlation was observed between junk food consumption and anaemia	High consumption of junk food is positively correlated with an increased risk of anaemia. Implementing awareness programs and counseling sessions has been shown to significantly improve knowledge and behaviors related to anaemia prevention. Therefore, combining dietary modifications with targeted educational interventions is essential for effectively reducing anaemia cases
Benash Altaf, et.al, 2017	The aim is to determine the frequency of anemia and to find its association with junk food among medical students	Cross-sectional study	Pakistan	N = 112 Boys—58 Girls—54 18–25 years	Data was collected through pre-designed proforma Data collected on age, BMI, junk food consumption, and dietary habits Dietary habits were also recorded on pre-designed proforma Haemoglobin levels checked using Sahil's method and automated analyser to assess anaemia	49.1% of students consumed junk food; 69.1% were anaemic A significant negative association was found between junk food consumption and hemoglobin levels (P value 0.009*) Further Regression analysis confirmed a negative association between junk food intake and haemoglobin levels	Anaemia is more prevalent among girls consuming junk food than boys, highlighting the significant negative association of junk food with anaemia
Jeong, Jaehoon, Cho, Younghoon In-Young Cho, Joonho Ahn, et.al 2022	To Investigate obesity's link to anemia in adolescents	Cross-sectional study	South Korea	N = 185,731 Boys −12,697 Girls- 173,033 10–21 years	Data collected from the 2007–2019 Korea National Health and Nutrition Examination Survey Health interviews were conducted by trained medical staff Health examinations and height and weight were performed in mobile examination centres Anaemia was measured according to WHO criteria	The risk of Anaemia is higher in obese early adolescents (OR 2.88; 95% CI 1.20–6.95) No significant relationship between obesity and anaemia was observed in adolescents overall, except in early adolescents aged 10–13, where obesity was linked to an increased risk of anaemia	Anaemia is a treatable condition that requires special attention in obese early adolescents, as they are at an increased risk
Ayub Hina et al., 2023	To evaluate dietary factors contributing to anemia among girls	cross-sectional	Pakistan	N = 130 Girls −130 12–16 years	Data on socioeconomic, demographic, and dietary factors collected via a questionnaire A Self-developed questionnaire was used for dietary information collection (i.e., dietary habits, food intake, vegetarian or non-vegetarian dietary patterns, consumption of junk food) The cyanmethemoglobin method was used for Hb estimation	82% of adolescent girls in urban and rural areas were anaemic (p = 0.001) Among rural girls, 43.84% had mild anaemia, and 13% had severe anaemia 70.7% of girls were underweight, 93% followed a mixed dietary pattern 44.1% reported irregular menstruation, 87.6% experienced menstruation lasting more than 5 days	Anaemia is a significant health issue, among girls especially in rural areas, where awareness of iron-rich diets is often limited. To effectively address anaemia among adolescents, it is essential to prepare the public health system to implement programs that raise awareness and promote the consumption of iron-rich foods
Sabbah, Haleama Al 2020	The present study assessed the associations of overweight, obesity and anaemia with selected lifestyle factors, total body fat and abdominal obesity among female university students	Cross-sectional study	United Arab Emirates	N = 251 Females – 251	A self-reported questionnaire was utilized for data collection The questionnaire included items on obesity, anaemia, and lifestyle Blood samples were collected using finger pricks Hb levels were measured with a Hemocue analyser for Hb level measurement Associations of lifestyle factors, total body fat, and abdominal obesity examined	29.3% of participants were classified as overweight or obese 18.1% of participants were diagnosed with anaemia 8.5% of participants exhibited abdominal obesity Total body fat percentage was highest among students with anaemia	The prevalence of anaemia and obesity/overweight among female university students highlights the need for health promotion and nutritional education programs. Implementing such initiatives is essential to enhance the overall health and well-being of this population
Sari P, Bestari. A, Astuti, R, Bestari D 2019	To determine the prevalence of anemia and its correlation between anemia and nutritional status among adolescent girls	Community-based Cross-sectional survey	Indonesia	N = 92 Girls—92 10–19 years	Nutritional status is classified by BMI categories according to WHO standards Haemoglobin concentration determined by cyanmethemoglobin method 24 h of dietary intake, through interview methods	26.09% of adolescent girls had anaemia, 73.91% had normal haemoglobin levels No significant correlation between nutritional status and anaemia among adolescent girls Anaemia linked to iron deficiency, dietary habits, and demographic factors	Nutritional status did not significantly contribute to anaemia in adolescent girls, as various factors can cause anaemia, such as dietary iron deficiency and sociodemographic characteristics

### Step 6: Stakeholder Consultation

We did not engage in stakeholder consultation in this review due to the consequence of time and financial constraints**.**

## Results

**Fig. 1:**
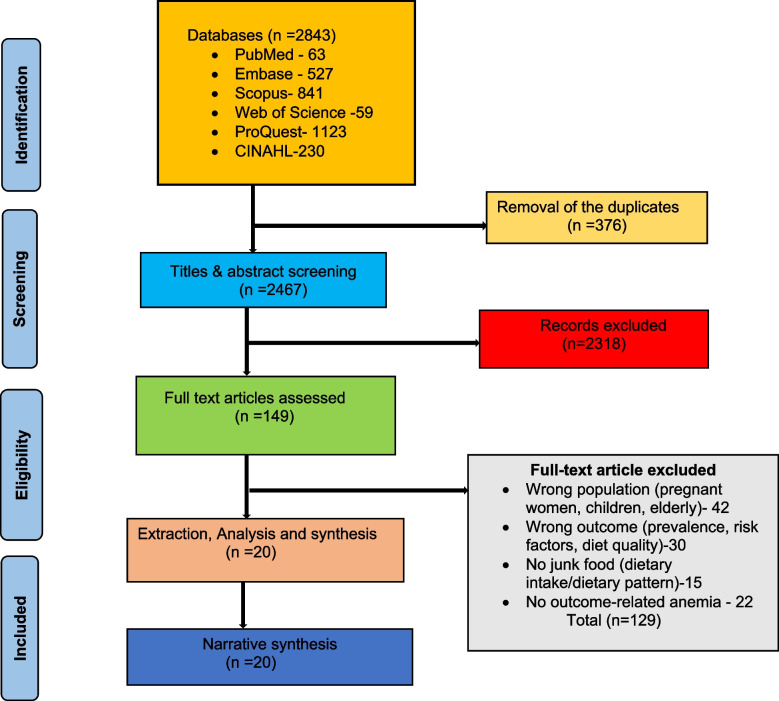
A PRISMA flowchart of the study selection process

Electronic searches were conducted on PubMed (MEDLINE) (*n* = 63), Web of Science (Clarivate) (*n* = 59), CINAHL(EBSCO) (*n* = 230), EMBASE (Elsevier) (*n* = 527), Scopus (*n* = 841), ProQuest (*n* = 1123), Of the 2843 records retrieved, 376 duplicates were removed using Rayyan. Further, 2467 articles were screened for Title-Abstract, and 146 articles were found eligible for full-text screening. Of the 146 articles, 20 were included for analysis, and others were excluded due to wrong publication type (*n* = 48) and wrong outcome (*n* = 8). The PRISMA flow diagram is presented in Fig. 1.Appendix 2 presents a list of records excluded during the full-text stage.

### Characteristics of Included Studies

#### Study settings

Four studies were conducted in Indonesia [19–22], five studies were conducted in India [23–27], five studies were conducted in Pakistan [28–32], and one study each was conducted in China [33], Mexico [34], Palestine [35], Taiwan [36], UAE [37] and South Korea [38].

### Study Designs

The data for this review were collated through cross-sectional studies and observational studies. However, there were few Randomized and non-randomized Controlled trials on this topic, and the authors had to exclude those studies since they did not meet the inclusion criteria. This review included data from cross-sectional observational studies. The characteristics of the included studies are detailed in Table 1.

The findings of our review is categorised as impact of junk foods on anaemia, types of the junk food taken, the frequency and consumption of junk food, other risk factors related to anaemia and additional findings from the studies.

### Impact of junk foods on anaemia

Numerous studies have demonstrated a link between the consumption of convenience foods, fast food, snacks, and beverages with a prevalence of anaemia, particularly among adolescents undergoing puberty [33]. A Western diet, which is high in energy, fat, and sugar, has been found to have a stronger association with anaemia compared to non-traditional breakfasts, which were inversely related to anaemia [34]. Another study highlighted a significant association (*p* < 0.05) between iron deficiency (ID), anaemia, iron deficiency anaemia (IDA), and dietary habits such as skipping breakfast and consuming large amounts of junk food. [35]. One study showed that there is a relationship between diet pattern and the incidence of anaemia, lower a person’s diet, higher the category of anaemia experienced also it was found that 88% of adolescent were of Poor eating patterns, they preferred it as fast food because it is easily available fast processing and was waiting time was less [19]. Another study revealed that female teenagers with insufficient iron intake were eight times more likely to develop anaemia compared to those with adequate iron intake. [21]. Additionally, in one study, a positive link was found between poor eating habits, specifically the frequent consumption of unhealthy, junk foods and anaemia, with most adolescents showing a preference for junk and fast foods. [29]. Also, another study revealed a positive correlation between intake of junk food and the risk of anaemia, which showed that higher levels of junk food consumption are linked to an increased likelihood of anaemia, while lower levels of consumption correspond to a reduced risk of the condition. [30] study also found that a significant portion of junk food consumers, specifically 69.1%, were found to be anaemic, with their haemoglobin levels notably lower compared to those who did not consume junk food [31]. Studies have demonstrated that a large proportion of adolescents regularly indulge in junk food at school, leading to a reduced consumption of eggs and fish [32] (Table 1).

### Type of junk foods

In our review, we found that the types of junk foods most frequently consumed by the adolescents included fried chicken, chicken soup, martabak, noodles, dumplings, fried foods, sempol, cilok, and pentol, with daily consumption being common. [19] Studies indicates that the snacks most frequently consumed by teenagers consist of meatballs, chicken noodles, fried foods, and donuts [21, 29] Additionally, a significant number of adolescents habitually consumed instant noodles, fried rice, and fried noodles [22], along with chocolate biscuits and candies [28]. One study identified dietary patterns related to anaemia and inflammation, characterized by a high intake of eggs, meat, organ meats, rice or flour products, fried rice or flour, sugary beverages, fried foods, and processed foods [36] A study observed that adolescents frequently consumed a Western—type diet characterized by industrialized sweet drinks, salty snacks, sandwiches, charcuterie [34]. (Table 1).

### Frequency of consumption of junk food and anaemia

Most adolescents consumed junk food daily, while fruits and vegetables were typically eaten only once a week or, for some, just 3–6 times a month [19]. A significant portion, 71.1%, consumed meat and meat products three times a week. [29]. One study found a significant relationship between anaemic status and their frequency of fast food consumption (*p* = 0.04*) [25]. Also in one study, the frequency of fast food consumption was inversely related to haemoglobin levels in female adolescents (*r* = −2.07; *p* = 0.025), indicating that the more often they consumed fast food, the lower the Hb levels tended to be [22]. A study has revealed a robust association between the consumption of junk food and anaemia in adolescents. Specifically, it was found that 81.5% of participants who consumed junk food on a bi-weekly basis and 96.6% of those who frequently skipped breakfast were affected with anaemia. [23]. Many adolescents had a frequent habit of consuming junk food [24] (Table 1).

### Risk factors

The highest risk for anaemia included being underweight [29, 30, 32, 33] with a higher prevalence observed in women compared to men. Other significant factors were age, gender, [36] parental education level [33], obesity [19], specifically those with obesity, central obesity, or who were underweight had an increased risk of anaemia [36]. Additionally studies such as nuclear family, high intake of junk foods, low intake of milk, eggs, green leafy vegetables [28], citrus fruits, lack of folic acid intake and menorrhagia [26], and current alcohol consumption, were associated with higher risk of anaemia [30].Obesity was notably linked to a greater risk of anaemia, particularly in early adolescents. [38], Adolescents consuming Junk food twice or more in a week were more over weight and at risk of overweight as compared to them who consumed junk food only once or rarely in a week. [23]. iron deficiency was also a significant risk factors for anaemia [36] (Table 1).

### Additional findings

*Sociodemographic characteristics*: Most of the studies in our review found that lower socio-economic background are significantly associated with anaemia (*p* < 0.01), [28] also study emphasized on other factors like parental education (*p* = 0.022), especially mothers education [35] unemployment [28], in one study it was evident that adolescents from higher socioeconomic class are more prone to overweight and obesity. Due to greater financial means to frequently purchase junk food. This increased the access to junk food can lead to overweight and obesity. [23]. One study have highlighted that anaemia, along with overweight, obesity and anaemia were more prevalent among female students who perceived their families having a moderate economic status. [37].

### Dietary patterns

Dietary patterns play a significant role in the prevalence of anaemia, particularly among adolescents low consumption of infrequent consumption of eggs, milk, green leafy vegetables such as spinach, and even indulgences chocolates, candies, biscuits, cakes have associated with increased risk of anaemia. [28] whereas in one study shows that food patterns like meat and eggs have been to be protective against anaemia. Studies have highlighted that decrease in meat consumption is likely to lead to anaemia, and diet lacking milk and beverages is significantly associated with anaemia [35]. Additionally study also showed that reduced intake of vegetable, fruit and meat is more likely to result in iron deficiency anaemia [35]. One study showed that dietary patterns characterized by high intakes of eggs, meat, organ meats, rice or flour products, fried foods, sugary beverages, and processed foods significantly increased the risk of anaemia. This pattern is linked with decreased levels of haemoglobin, haematocrit, and red blood cells, while it elevates white blood cells and C-reactive protein levels. [36]. Moreover, in one study specific dietary habits have been closely associated with varying degrees of anaemia. for instance women who did not consume green leafy vegetables had mild to moderate anaemia, women who drank tea or coffee immediately after meal had mild anaemia and women eating junk foods and less fruit intake had mild to moderate anaemia [27] A study found a strong correlation between skipping breakfast and anaemia among adolescents. Those who skipped breakfast twice or more per week were significantly more likely to be anaemic compared to those who skipped breakfast only once or rarely in a week. This relationship highlights the impact of breakfast habits on the prevalence of anaemia. [23], In one study. Anaemia appears to be more prevalent among vegetarians than non-vegetarians, particularly among those with a predominantly rice-based diet (‘r’ = 0.871). There was increased association on consumption of tea and coffee post- meals (r = 0.892). [24] and the occurrence of anaemia. Furthermore, western patterns of diet have been linked with stunting. [34] in one studies showed that skipped meals, such as breakfast, or lunch are more likely to lead to anaemia. [35].

*Lifestyle and Physical activity*: One study showed the relationship between alcohol intake and a lack of physical was positively correlated with the occurrence of anaemia. Factors such as age, gender, hypertension, and both smoking and alcohol use, which were significantly related to anaemia. [36] A study revealed a larger number of patient with iron deficiency anaemia had moderate physical activity. [30].

*Obesity*: The prevalence of obesity was high among the adolescent who consumed western pattern of diet. Both Western and plant-based patterns were linked to overweight and obesity as well as at least one indicator of undernutrition [34]. Additionally, the consumption of fast food and the habit of skipping breakfast increased as adolescents transitioned into adulthood, with these dietary behaviours being associated with greater weight gain during this period. [23] In one study approximately one-third of female university students were identified as overweight or obese, with a prevalence of having abdominal obesity and anaemia. The Majority of these students reported irregular eating patterns, especially among those who were obese or overweight. Also the study found that both the Western and plant-based patterns were simultaneously associated with overweight–obesity and at least one indicator of undernutrition. [34].

## Discussion

The present study aimed to explore the impact of junk food on the prevalence, severity of anaemia, particularly among adolescents. Our findings reveal a significant association between frequent consumption of junk food and an increased risk of anaemia, as evidenced by lower haemoglobin levels and a higher incidence of iron deficiency. Also some additional findings like other factors role of socio-demographic factors, dietary patterns, obesity, lifestyle and physical activity were linked with anaemia.

### Impact of junk food on anaemia

Numerous studies have found that there is a link between junk food consumption and anaemia one of the most common type of anaemia is iron deficiency anaemia and megaloblastic anaemia in young population [39]. Most of the adolescents prefer western type of diet [40] which increases the incidence of anaemia. A systematic review seven key topics on nutrient adequacy, fruit and vegetable consumption, water and beverage intake, sodium (Na) intake, breakfast habits, snacking frequency, and Western fast food consumption revealed the following adolescents tend to consume insufficient amounts of protein, fruits, and vegetables, while their intake of sodium and Western fast food is excessively high. [41]. The study found a significant correlation between breakfast habits and the incidence of anaemia among adolescents. Results showed that students who skip breakfast have a one or two –times higher risk of developing anaemia compared to those who regularly eat breakfast. Statistical analysis revealed that (*p* = 0.036) a significant relationship between skipping breakfast and the increased risk of anaemia. [42]. Whereas in this review skipping breakfast were directly related to anaemia.

Junk food typically contains high levels of trans fats, salt, and sugar, but is low in essential nutrients, contributing to poor nutritional outcomes usually Today, many foods are made with extra sugar and fat, making them even less healthy [43]. Thus consumption of junk food are high among adolescents [44]. These results align with the hypothesis that diets high in processed foods, which are typically low in essential nutrients like iron, contribute to the development of anaemia. A study conducted have shown that the iron content in fried Tempe and noodle showed a significant difference with *p* > 0.05, while the iron content in fried chicken in the two school groups did not show different results. However, the percentage contribution of iron to the Nutrition Adequacy Rate (RDA) was minimal [45], also exposure to junk food related content are linked to heightened sensations of hunger, stress, sadness, and fatigue, along with an increased desire for salty, savoury, and fatty foods [46]. Most of junk foods consumed by the adolescents are soups, noodles, fried foods, meat balls sweet drinks, salty snacks, sandwiches. These findings are similar to a study where fast food consumed frequently by 36% of individuals, 12% reported regular consumption of sugar-sweetened beverages. The items most often consumed included salty snacks (77%), and regular soda (77%), [47] and also most of the adolescent they consumed inadequate fruits and vegetables [48] which also a similar findings in this review.

The findings of this review suggest a significant association between the frequency of fast-food consumption and the prevalence of anaemia among adolescents. The study indicate that a majority of adolescents consume junk food daily, which correlates with lower haemoglobin levels. Interestingly, while meat and meat products were typically consumed around three times per week, the intake of junk food had a more pronounced negative impact on haemoglobin levels. Adolescents with higher junk food consumption tended to have lower haemoglobin levels, increasing their risk of anaemia.

Moreover, the review highlights that adolescent who frequently skipped breakfast was particularly vulnerable to anaemia. This aligns with other studies that observed similar trends, where the frequency of junk food consumption less than three times a week or up to three times a week was linked to an increased risk of anaemia [49]. Furthermore, additional research supports these findings, showing that girls who routinely skipped meals, especially breakfast, were more likely to develop anaemia [50]. These patterns underscore the critical role of dietary habits in adolescent health, particularly in relation to anaemia prevention.

In addition to the observed dietary patterns, there are important biological mechanisms by which junk food consumption contributes to iron deficiency anaemia. Diets dominated by energy-dense, nutrient-poor foods lack essential micronutrients such as vitamin C, which facilitates non-heme iron absorption, and instead contain inhibitors like phytates, calcium, and polyphenols that hinder iron bioavailability. Additionally, frequent consumption of high-fat, sugary, and processed foods has been linked to low-grade systemic inflammation, which can stimulate the hepatic synthesis of hepcidin—a regulatory hormone that inhibits intestinal iron absorption and iron release from macrophages and liver stores [36] Elevated hepcidin levels result in functional iron deficiency, even when total body iron stores may appear sufficient. Furthermore, junk foods often displace iron-rich whole foods such as meats, legumes, eggs, and green leafy vegetables [35, 36]. One study in this review also observed that dietary patterns high in fried and processed foods, sugary beverages, and refined carbohydrates were associated with reduced haemoglobin, haematocrit, and red blood cell levels, and increased levels of C-reactive protein—a marker of inflammation [36]. These biological interactions underscore the importance of not only reducing junk food consumption but also promoting balanced diets to prevent and manage anaemia, particularly among adolescents.

### Role of Socio-demographic factors

Also, some of the additional findings of this reviews are age, gender as critical factors. Specifically, being females and younger age is consistently associated with higher anaemia risk. This is supported by findings that girls, particularly those who menstruate, are at increased risk. [2].Key risk factors identified include being underweight, particularly in girls, with higher prevalence among women compared to men. Obesity including central obesity and being underweight, also significantly increases the risk of anaemia. Other notable factors include age, gender, parental education level, and iron deficiency. Similar factors were found in other studies [1, 2].

### Dietary Patterns and Nutrient Deficiency

Dietary practices play a crucial role, with high junk food consumption and low intake of nutritious foods like milk, eggs, green leafy vegetables, and citrus fruits contributing to anaemia risk. A study finding showed that the daily intake of nutritious foods among adolescent girls was low, with only 16% consuming dairy, 46% eating meats, 44% eating fruits, and 37% eating vegetables. In contrast, energy-dense, nutrient-poor options like sweet snacks, salty snacks, fast foods, and sugar-sweetened beverages were consumed four to six times per week by girls, respectively. Additionally, 40% of the girls reported that they skipped breakfast. Thus many studies have shown that dietary pattern plays an important role in developing anaemia where similar findings have been found in this review [51].

### Socioeconomic and Educational Influence

Also, Socioeconomic status and educational level are indirect but influential risk factors, as lower socioeconomic status and educational attainment are associated with a higher risk of anemia risk [28, 35, 37].

### Strengths and Limitations

There is a dearth of synthesized literature on the consumption of junk on anaemia. This review may be one of the first to attempt to know how junk foods contribute to the development of anaemia among adolescent girls. These studies on dietary patterns and dietary practices, make their findings highly applicable to everyday scenario. This review covers a broad range of junk foods and their potential impacts on anaemia, providing comprehensive insights into dietary patterns. Various electronic databases have been utilized to facilitate a thorough search of the literature. the studies have been scrutinized in a greater detail to clarify several aspects concerning the consumption of junk foods and it links to anaemia. The study offers insights to practitioners, policymakers, researchers, and adolescent groups. This review has not included qualitative studies in its literature and we acknowledge this as a significant limitation. Some studies use longitudinal designs to track changes in diet and anaemia over time, offering valuable insights into causal relationships. Many studies rely on self-reported dietary data which can be inaccurate due to recall bias or misinterpretation. Most of the studies are cross-sectional designs are often used in research, as they can indicate association; however, they do not establish a causal relationship between junk food consumption and anaemia. Factors like overall diet quality, socioeconomic status, and other health conditions might confound the relationship between junk food consumption and anaemia. Results may not be generalizable to all populations or age groups, particularly if the study sample is specific or limited different studies may define"junk food"and"anaemia"differently, leading to inconsistencies and difficulties in comparing findings across studies. Furthermore, the study did not account for other factors that may influence anaemia, such as genetic predisposition, underlying health conditions, or socioeconomic status. Future research should focus on Randomized Controlled trials and longitudinal studies to better establish the causal relationship between junk food consumption and anaemia. Additionally, studies that investigate the specific components of junk food that contribute to anaemia, as well as the effectiveness of dietary interventions in reducing anaemia risk, would be valuable.

### Implications

This study highlights the critical need for more comprehensive research to fully understand the relationship between junk food consumption and anaemia in adolescents. The current evidence suggests a potential link between poor dietary habits, including high intake of fast food and low consumption of nutritious foods like fruits, vegetables, and iron-rich items, and the development of anaemia. Addressing these modifiable risk factors could be essential in developing effective prevention and management strategies for anaemia among adolescents. Moreover, this study aligns with global health priorities, such as Sustainable Development Goal (SDG) 3, which focuses on promoting health and well-being, particularly by addressing adolescent health and preventing anaemia-related complications. Additionally, the findings support broader initiatives aimed at ending hunger, achieving food security, and improving nutrition.

## Conclusion

The study concludes that there is emerging evidence suggesting a link between junk food consumption and the prevalence of anaemia among adolescents, particularly in developing countries. While some studies indicate significant associations, others do not, highlighting the need for more comprehensive research to fully understand this relationship. The findings emphasize the importance of addressing modifiable dietary risk factors, such as the intake of fast food and poor eating habits, in the prevention and management of anaemia in adolescents. The mixed results from the analyzed studies indicate that more in-depth research is needed to clarify this relationship. Understanding how dietary habits, particularly the consumption of energy-dense, nutrient-poor foods, contribute to anaemia is crucial for developing targeted interventions. The research highlights the necessity of tackling the dietary risk factors within the framework of comprehensive public health initiatives aimed at decreasing the incidence of anaemia among adolescents and enhancing their overall status. This research supports global health initiatives, particularly Sustainable Development Goal (SDG) 3, which focuses on promoting well-being and addressing adolescent health, as well as efforts to end hunger, achieve food security, and improve nutrition.

## Supplementary Information

Additional file 1.

## Data Availability

Data is provided within the manuscript or supplementary information files.
