# Supplementary material for: Mapping evidence on the impact of junk food on anaemia among adolescent and adult population: a scoping review
Source: BMC Nutr. 2025 May 14;11:96. doi: 10.1186/s40795-025-01079-1 (PMC12079900; doi:10.1186/s40795-025-01079-1)
Supplement: Supplementary file 1 — Additional file 1. [file 40795_2025_1079_MOESM1_ESM.docx]

**Appendix 1**

**Pubmed 63**

(("Eating"[MeSH Terms] OR "Dietary intake"[All Fields] OR ("Eating"[MeSH Terms] OR "Eating"[All Fields]) OR ("intake"[All Fields] OR "intake s"[All Fields] OR "intakes"[All Fields]) OR ("consumptions"[All Fields] OR "economics"[MeSH Terms] OR "economics"[All Fields] OR "consumption"[All Fields])) AND ("Fast Foods"[MeSH Terms] OR (("fasting"[MeSH Terms] OR "fasting"[All Fields] OR "fast"[All Fields]) AND "food*"[All Fields]) OR "Snacks"[MeSH Terms] OR ("snacked"[All Fields] OR "Snacks"[MeSH Terms] OR "Snacks"[All Fields] OR "snack"[All Fields] OR "snacking"[All Fields]) OR "food, preserved*"[MeSH Terms] OR "preserved food*"[All Fields] OR "food, processed*"[MeSH Terms] OR "frozen foods*"[MeSH Terms] OR "candy*"[MeSH Terms] OR "junk food*"[All Fields] OR ("canned"[All Fields] OR "canning"[All Fields]) OR "convenience food*"[All Fields] OR "Ultraprocessed foods"[All Fields] OR "ultra processed foods*"[All Fields]) AND ("Hemoglobins"[MeSH Terms] OR "haemoglobin*"[All Fields] OR "Anemia"[MeSH Terms] OR "anemia, iron deficiency"[MeSH Terms] OR "iron deficiency anaemia"[All Fields] OR ("anaemia"[All Fields] OR "Anemia"[MeSH Terms] OR "Anemia"[All Fields] OR "anaemias"[All Fields] OR "anemias"[All Fields])) AND ("Adolescent"[MeSH Terms] OR "adolescent*"[All Fields] OR ("adolescences"[All Fields] OR "adolescency"[All Fields] OR "Adolescent"[MeSH Terms] OR "Adolescent"[All Fields] OR "adolescence"[All Fields] OR "adolescents"[All Fields] OR "adolescent s"[All Fields]) OR ("Adolescent"[MeSH Terms] OR "Adolescent"[All Fields] OR "teen"[All Fields]) OR "teen*"[All Fields] OR "teenager*"[All Fields] OR ("Adolescent"[MeSH Terms] OR "Adolescent"[All Fields] OR "teenage"[All Fields] OR "teenager"[All Fields] OR "teenagers"[All Fields] OR "teenaged"[All Fields] OR "teenager s"[All Fields] OR "teenages"[All Fields]) OR "youth*"[All Fields] OR ("Adolescent"[MeSH Terms] OR "Adolescent"[All Fields] OR "youth"[All Fields] OR "youths"[All Fields] OR "youth s"[All Fields]) OR ("young"[All Fields] OR "youngs"[All Fields]) OR "youngster*"[All Fields] OR ("youthful"[All Fields] OR "youthfulness"[All Fields]))) AND ((y_10[Filter]) AND (humans[Filter]))

Embase 527

Query('adolescent'/exp OR 'adolescent' OR 'adolescence'/exp OR 'adolescence' OR 'juvenile'/exp OR 'juvenile' OR teens OR youngster OR teen OR 'teenager'/exp OR teenager OR 'youth'/exp OR youth) AND ('eating' OR 'consumption' OR 'food intake'/exp OR 'food intake' OR 'eating'/exp OR eating OR 'consumption'/exp OR consumption OR 'food'/exp OR food) AND ('fast food'/exp OR 'fast food' OR 'junk food'/exp OR 'junk food' OR 'processed food'/exp OR 'processed food' OR 'frozen food'/exp OR 'frozen food' OR 'convenience food'/exp OR 'convenience food' OR 'fast' OR 'fast'/exp OR fast OR 'candy' OR 'candy'/exp OR candy OR 'convenience' OR 'convenience'/exp OR convenience) AND ('hemoglobin'/exp OR 'hemoglobin' OR 'hemoglobin variant'/exp OR 'hemoglobin variant' OR 'anemia'/exp OR 'anemia' OR 'iron deficiency anemia'/exp OR 'iron deficiency anemia' OR 'haemoglobin'/exp OR haemoglobin OR 'iron'/exp OR iron OR 'anaemia'/exp OR anaemia) AND [2014-2024]/py

Proquest (1,123)

**((anaemia OR (iron deficiency anaemia) OR haemoglobin) AND (Adolescents OR Adolescence OR teens OR teenagers OR young OR youngsters OR Youth) AND (Eating OR (dietary intake) OR intake OR Consumption) AND ((ultraprocessed food) OR (Fast Food) OR Snacks OR snacking OR (Food Preservatives) OR (preserved food) OR (frozen food) OR Candy OR (junk food) OR (canned food OR Convenience food))) AND (at.exact("Article") AND subt.exact(("diet" OR "food" OR "nutrition" OR "hemoglobin" OR "nutrition research" OR "womens health" OR "public health" OR "iron" OR "anemia" OR "clinical trials") NOT ("diabetes" OR "metabolism" OR "obesity" OR "proteins" OR "glucose" OR "patients" OR "body mass index" OR "age" OR "diabetes mellitus" OR "mortality" OR "inflammation" OR "insulin" OR "risk factors" OR "insulin resistance" OR "questionnaires" OR "blood pressure" OR "cardiovascular disease" OR "exercise" OR "lipids" OR "hypertension" OR "physiology" OR "oxidative stress" OR "children" OR "cholesterol" OR "metabolites" OR "antioxidants" OR "population" OR "enzymes" OR "malnutrition" OR "body weight" OR "laboratories" OR "biomarkers" OR "chronic illnesses" OR "health risks" OR "studies")) AND la.exact("ENG") AND stype.exact("Scholarly Journals") AND pd(20140721-20240721))**

**Scopus 841**

( ALL ( "Eating" OR "Dietary intake" OR eating OR intake OR consumption ) ) AND ( ALL ( "Fast Foods" OR fast AND food* OR "Snacks" OR snacking OR "Food, Preserved*" OR "Preserved food*" OR "Food, Processed*" OR "Frozen Foods*" OR "Candy*" OR "junk food*" OR canned OR "convenience food*" OR "Ultraprocessed foods" OR "Ultra-processed foods*" ) ) AND ( ALL ( haemoglobin* OR "Anemia, Iron-Deficiency" OR "iron deficiency anaemia*" OR anaemia ) ) AND ( ALL ( "Adolescent" OR adolescent* OR adolescence OR teen OR teen* OR teenager* OR teenage OR youth* OR youth OR young OR youngster* OR youthful ) ) AND PUBYEAR > 2013 AND PUBYEAR < 2024 AND ( LIMIT-TO ( EXACTKEYWORD , "Human" ) ) AND ( LIMIT-TO ( LANGUAGE , "English" ) ) AND ( LIMIT-TO ( SRCTYPE , "j" ) )
